# The effect of low donor-to-recipient body weight ratio on graft survival after dual kidney transplantation from pediatric deceased donors

**DOI:** 10.1080/0886022X.2025.2454968

**Published:** 2025-01-22

**Authors:** Mingchuan Huang, Shenghui Wu, Pengfei Gao, Li Zhou, Qian Fu, Chenglin Wu, Huanxi Zhang, Yitao Zheng, Xiaojun Su, Wenrui Wu, Jinghong Tan, Qiang Zhang, Pei Xia, Zhe Xu, Longshan Liu, Jun Li, Changxi Wang

**Affiliations:** a Department of Pediatric Surgery, First Affiliated Hospital of Sun Yat-sen University, Guangzhou, China; b Organ Transplantation Center, First Affiliated Hospital of Sun Yat-sen University, Guangzhou, China; c First Affiliated Hospital, Nanjing Medical University, Nanjing, China; d Kidney Transplantation Department, The Third People’s Hospital of Shenzhen, Shenzhen, China; e Department of Pathology, First Affiliated Hospital of Sun Yat-sen University, Guangzhou, China; f Guangdong Provincial Key Laboratory of Organ Donation and Transplant Immunology, The First Affiliated Hospital, Sun Yat-sen University, Guangzhou, China; g Organ Transplant Center, Guangdong Provincial International Cooperation Base of Science and Technology (Organ Transplantation), Guangzhou, China

**Keywords:** Dual kidney transplant, pediatric donor, body weight ratio, survival analysis, surgical procedure

## Abstract

**Background:**

Dual kidney transplantation (DKT) from small pediatric donors, either en-bloc or split dual kidney transplantation, contributes to mitigating organ scarcity. This study investigates the prognosis of DKT from pediatric deceased donors, and influencing factors.

**Method:**

A retrospective study included recipients who underwent DKT from pediatric donors between 2012 and 2022. Recipients were categorized into low mismatch (BWLM, *n* = 30) and high mismatch (BWHM, *n* = 10) groups based on donor-recipient weight ratio of 1:10. Outcome encompassed recipient and graft survival, renal function, and adverse events.

**Result:**

Forty recipients were included. The average follow-up period was 54.6 months. The 1, 3, and 5-year patient survival were 97.4%, with no significant differences between en-bloc and split dual kidney transplantation or between BWLM and BWHM groups. The graft survival at 1, 3, and 5 years were 89.9%, the graft survival of BWHM group was lower than BWLM group (70% vs 96.7%, *p* = 0.039). The average eGFR at 1, 3, and 5 years postoperatively were (78.93 ± 25.23), (83.82 ± 32.4), and (85.92 ± 37.08) mL/min/1.73 m^2^, respectively. The BWHM group also experienced higher rates of graft-related surgical complications (*p* = 0.006) and urinary tract surgical complications (*p* = 0.042).

**Conclusion:**

DKT from pediatric donors yields favorable outcomes, with similar graft survival and complication rates across surgical subgroups. However, significant donor-recipient weight mismatch, particularly when the ratio is less than 1:10, may contribute to increased surgical complications and poorer graft survival. Efforts to minimize extreme weight mismatch are recommended to optimize outcomes.

## Introduction

Kidney transplantation is considered the most effective treatment for patients with end-stage renal disease (ESRD), and the advantages over dialysis are particularly pronounced when performed earlier [1,2]. With the gradual improvement and promotion of donation policies, pediatric donors have become an important source for expanding the donor pool, resulting in a heightened utilization of pediatric kidneys [3–6]. This has resulted in more transplantation opportunities for candidates on the waiting list, thereby alleviating the organ shortage and addressing the growing gap between supply and demand. Currently, pediatric donors account for over 10% of the overall donor pool, and the overall prognosis is deemed satisfactory [3,7–11].

However, for infant donors with very low age and body weight, the single kidney transplantation (SKT) procedure poses challenges in terms of matching and may result in complications due to insufficient functional renal units and high perfusion injury. With the accumulation of experience in preoperative donor-recipient evaluation, improvements in surgical techniques, and meticulous perioperative management, pediatric dual kidney transplantation (DKT) has emerged as a viable option for patients with end-stage renal disease (ESRD), offering favorable long-term outcomes. DKT involves the simultaneous transplantation of both kidneys from the same donor into a single recipient, ensuring an adequate number of functional renal units [12–15]. The commonly used surgical techniques include en-bloc kidney transplantation (EBKT), which refers to the surgical technique of anastomosing the inferior vena cava and aorta patch of both donor kidneys to the venous and arterial conduits of a single recipient, and split dual kidney transplantation (SDKT), which involves separating the two kidneys at the back table after dividing the abdominal aortic patch and inferior vena cava, followed by two separate kidney transplantations on the same side (usually) in the recipient. However, to date, only limited studies have been conducted elucidating the disparities in prognosis between these two surgical procedures.

Smaller kidneys inherently have finer blood vessels, a characteristic that can elevate the risk of post-transplant complications such as thrombosis, which may ultimately lead to graft failure. This risk is particularly pronounced in pediatric kidney transplants, where the donor-recipient weight ratio plays a critical role in influencing outcomes. When there is a significant mismatch in body weight between donor and recipient, it can result in suboptimal graft function and an increased likelihood of postoperative complications. Studies have linked such mismatches to conditions like hyperfiltration injury, proteinuria, and thrombosis, all of which compromise long-term graft survival and patient health. Despite these findings, the optimal criteria for matching donor and recipient weights remain undetermined, highlighting a critical area for further research to enhance transplant success rates and reduce complication risks [16–19]. Because of the potential technical difficulties associated with these challenges, some transplant centers may choose to discard these kidneys. Consequently, kidneys from donors with very low body weight are often underutilized [3,20]. There are few comparative studies regarding the distinction between EBKT and SDKT. Similarly, studies examining the factors impacting the prognosis of DKT remain scarce. Therefore, this study aims to investigate the long-term prognosis of DKT from pediatric deceased donors and identify anthropometric indicators that can assist clinicians in making more informed decisions.

## Patients and methods

### Study design

This single-center retrospective study included recipients who underwent DKT from pediatric deceased donors between January 2012 and December 2022 at the First Affiliated Hospital of Sun Yat-sen University. Inclusion Criteria: Recipients who underwent same-donor dual kidney transplantation at the First Affiliated Hospital of Sun Yat-sen University; Recipients who received kidneys from deceased donors; Donors who were under 18 years old at the time of organ donation; Patients who underwent regular follow-up at our hospital after transplantation. Exclusion Criteria: Recipients who underwent combined organ transplantation; Patients who were unable to meet the minimum follow-up period of at least 6 months postoperatively. This study was approved by the Ethics Committee of The First Affiliated Hospital of Sun Yat-sen University (No. [2022]013).

### Study arms (groups)

All enrolled patients are divided into two cohorts based on the body weight ratio (BWR) of the donor and recipient, calculated by dividing the recipient’s weight by the donor’s body weight. The cohorts are stratified according to the first quartile (Q1), median, and third quartile of the BWR data, resulting in the formation of the donor-recipient body weight low mismatch group and donor-recipient body weight high mismatch group. A comparison is conducted between the two groups in terms of patient and graft survival, postoperative glomerular filtration rate, and postoperative complications to explore the impact of different levels of BWR on allograft outcomes. In consideration of the fact that our recipients undergo two different surgical procedures, in order to mitigate the potential impact of different procedures on outcomes, we also investigate the effects of these two procedures on the overall prognosis.

### Surgical procedures and perioperative management

The kidneys were procured en bloc and were prepared on the back table for transplantation. The surgical incision of right extraperitoneal approach to the iliac fossa was preferred. For EBKT, the distal end of aorta and inferior vena cava was ligated or continuously sutured, and the proximal end of aorta and inferior vena cava was separately end-to-side anastomosed to the recipient′s external iliac artery and external iliac vein with 6-0 or 7-0 vascular sutures. For SDKT, the two kidneys were split and prepared on the back table. The renal artery and vein of the two kidneys were subsequently end-to-side anastomosed to the recipient′s external iliac artery and vein with 7-0 vascular sutures. Both ureters were separately anastomosed to the recipient′s bladder. Two 4-F or 4.7-F ureter stent were placed in the ureters during transplantation and was removed at 2-4 week after transplantation if there was no urinary leakage or hemorrhage around bladder anastomosis.

All recipients received anti-thymocyte globulin or anti-CD25 monoclonal antibodies as induction therapy. Early maintenance immunosuppression regimens consisted of tacrolimus, mycophenolic mofetil or enteric-coated mycophenolic sodium and corticosteroids. Low molecular weight heparin (50-100 IU/kg/day) was daily administered as anticoagulation for 5-7 days after transplantation, and clopidogrel bisulfate (1-3 mg/kg/day) was subsequently used of 3-6 months. Anticoagulation is discontinued if there are signs of increased perirenal or intrarenal hemorrhage in the transplanted kidney. At the end of the arterial anastomosis during transplantation, papaverine was injected into the renal artery, and lidocaine was sprayed on the renal hilum to alleviate arterial spasms. Papaverine (30-60 mg/day) was then intravenously infused for 3-5 days after transplantation. The perioperative systolic blood pressure of the recipient was maintained at 110-130 mmHg according to the donor kidneys.

### Definitions of terms

Graft survival was defined as the requirement for re-transplantation, graft nephrectomy, irreversible return to dialysis, or patient death with a functioning graft. Graft-related surgical complications were defined as the occurrence of vascular thrombosis of the transplanted kidney, graft rupture with hemorrhage, perinephric hematoma, and renal artery stenosis. Urinary tract surgical complications were defined as urinary fistula and ureteral stenosis. Delayed graft function (DGF) was characterized by the necessity of dialysis within the first postoperative week. Renal biopsies were classified according to the Banff criteria by local pathologists. Graft function was assessed by measuring serum creatinine (SCr) levels and calculating the estimated glomerular filtration rate (eGFR). The eGFR was determined using the Modification of Diet in Renal Disease (MDRD) equation for patients older than 16 years, and the Schwartz equation for those aged 16 years or younger, both based on SCr levels.

### Statistical analysis

In the descriptive statistical analysis, quantitative variables that adhered to a normal distribution were expressed using the mean and standard deviation. If the data did not adhere to a normal distribution, median and interquartile range were utilized. Qualitative variables were presented as case numbers, frequencies, and percentages. When comparing baseline data and clinical outcomes, for continuous variables, an analysis of variance (ANOVA) test was conducted if the data adhered to a normal distribution and met the assumption of homogeneity of variances. If the data were skewed or did not adhere to the assumption of homogeneity of variances, the Mann-Whitney U test was employed. For categorical variables, the chi-square test or Fisher’s exact test was utilized. The Kaplan-Meier method was employed for analyzing patient and graft survival, while the log-rank test was utilized to compare survival rates between groups. All tests were two-tailed, and statistical significance was defined as *p* < 0.05. The statistical analysis was performed using IBM SPSS Statistics for Windows (version 27.0, IBM Corp.), GraphPad Prism for Windows (version 9.5.0, GraphPad Software), and R software for Windows (version 4.3.3, www.r-project.org).

## Results

### Population characteristics

According to the inclusion and exclusion criteria, a total of 40 DKT recipients from pediatric deceased donors were enrolled in this study. Among the 40 pediatric donors, 28 (70%) were male and 12 (30%) were female, with a median age of 4.5 months (ranging from 2 days to 43 months). Furthermore, 5 donors were under 1 month old, 10 were aged between 1 and 3 months, 7 were aged between 3 and 6 months, 8 were aged between 6 and 12 months, 7 were aged between 12 and 24 months, and 3 were aged between 24 and 48 months. The median weight of the donors was 6.25 kg (ranging from 3.9 kg to 9.2 kg), with the minimum donor body weight being 2.1 kg and the maximum donor body weight being 15 kg.

Among the 40 DKT recipients, 33 (82.5%) underwent EBKT procedures, and 7 (17.5%) underwent SDKT procedures. Consequently, the recipients were categorized into two distinct groups based on these surgical techniques, and their demographic characteristics have been presented in Table 1. With the exception of the donor type, no notable disparities were observed between these two groups. It is worth noting that among the 14 recipients (35%) who were less than 18 years of age, 4 patients underwent SDKT, constituting a substantial proportion of 57.14% (4 out of 7) within the SDKT group.

**Table 1.** The demographics and clinical characteristics of kidney transplant recipients and donors between EBKT and SDKT group.

Variables	Total (*n* = 40)	EBKT (*n* = 33)	SDKT (*n* = 7)	P
**Donor**				
Age (months), Median (Q1, Q3)	4.5 (2.0, 10.5)	6.0 (2.0, 12.0)	3.0 (2.5, 5.5)	0.326
Weight (kg), Median (Q1, Q3)	6.3 (3.9, 9.2)	6.5 (3.6, 10.0)	6.0 (5.2, 8.3)	0.957
EGFR (ml/min ·1.73 m^2^), Median (Q1, Q3)	54.7 (23.9, 102.9)	54.7 (26.9, 102.9)	50.0 (20.9, 103.7)	0.922
Donor type, n (%)				**0.039**
DBD	14 (35)	9 (27)	5 (71)	
DCD*	26 (65)	24 (73)	2 (29)	
**Recipients**				
Sex, n (%)				0.427
Female	22 (55)	17 (52)	5 (71)	
Male	18 (45)	16 (48)	2 (29)	
Age (years), Median (Q1, Q3)	29.3 (15.7, 38.0)	30.1 (15.9, 37.9)	16.8 (13.8, 34.6)	0.463
Weight (kg), Median (Q1, Q3)	43.3 (35.0, 54.3)	45.0 (35.1, 55.1)	38.0 (34.3, 44.8)	0.336
BWR, Median (Q1, Q3)	0.14 (0.1, 0.22)	0.13 (0.09, 0.22)	0.16 (0.11, 0.21)	0.601
Follow-up time (months), Mean ± SD	54.6 ± 37.4	57.0 ± 38.1	43.4 ± 34.5	0.389
CIT (h), Median (Q1, Q3)	11.8 (8.0, 18.6)	12.0 (10.0, 18.5)	8.0 (5.8, 15.8)	0.212
Induction, n (%)				0.128
ATG	32 (80)	28 (85)	4 (57)	
Basiliximab	8 (20)	5 (15)	3 (43)	
CNI, n (%)				1
FK506	40 (100)	33 (100)	7 (100)	
MPA, n (%)				0.068
Mycophenolate acid	15 (38)	10 (30)	5 (71)	
Mycophenolate mofetil	25 (62)	23 (70)	2 (29)	
Primary disease, n (%)				0.265
Glomerulonephritis	26 (65)	23 (70)	3 (43)	
IgA nephropathy	5 (12.5)	4 (12)	1 (14)	
Congenital or hereditary diseases	6 (15)	4 (12)	2 (29)	
Other	3 (7.5)	2 (6)	1 (14)	

EBKT, en-bloc kidney transplantation; SDKT, split dual kidney transplantation; EGFR, Estimated glomerular filtration rate; BWR, body weight ratio (donor/recipient); SD, standard deviation; CIT, cold ischemia time; ATG: antithymocyte globulin; CNI: calcineurin inhibitor; MPA: mycophenolic acid.

* According to the donor type standards published by Chinese scholars, the DCD referred to in this study includes Donation after Brain Death followed by Circulatory Death (DBCD), which differs from the broader definition of DCD used internationally [21].

The body weight ratio between donors and recipients was analyzed. The values for the first quartile, median, and third quartile were found to be 0.1, 0.14, and 0.22, respectively. Based on these criteria, the 40 recipients were divided into two groups. Significant differences were observed when using the first quartile for grouping comparison. Therefore, only the results from this analysis are presented in the main text. Results obtained using the median and third quartile will be included in the supplementary materials (see Supplementary). Recipients with a body weight ratio greater than 0.1 were categorized into the body weight low mismatch group (BWLM, *n* = 30), whereas those with a body weight ratio less than 0.1 were classified into the body weight high mismatch group (BWHM, *n* = 10). No significant differences were observed in the demographic characteristics of the recipients between the two groups (Table 2). However, concerning donors, the older age, lower body weight, and glomerular filtration rate of the kidneys were notably lower in the high difference mismatch group compared to the low difference group.

**Table 2.** The demographics and clinical characteristics of kidney transplant recipients and donors between BWLM and BWHM group.

Variables	Total (*n* = 40)	BWHM (*n* = 10)	BWLM (*n* = 30)	P
**Donors**				
Age (months), Median (Q1, Q3)	4.5 (2.0, 10.5)	1.5 (1.0, 3.75)	7.0 (3.1, 12)	**0.003**
Weight (kg), Median (Q1, Q3)	6.3 (3.9, 9.2)	3.3 (3.0, 3.6)	7.7 (5.4, 10)	**< 0.001**
EGFR (ml/min ·1.73 m2), Median (Q1, Q3)	54.7 (23.9, 102.9)	32.0 (23.0, 39.3)	75.7 (25.4, 110.0)	**0.035**
Donor type, n (%)				1
DBD	14 (35)	3 (30)	11 (37)	
DCD*	26 (65)	7 (70)	19 (63)	
**Recipients**				
Sex, n (%)				0.3
Female	22 (55)	4 (40)	18 (60)	
Male	18 (45)	6 (60)	12 (40)	
Age (years), Median (Q1, Q3)	29.3 (15.7, 38.0)	29.3 (17.4, 31.4)	29.3 (14.4, 38.6)	0.939
Weight (kg), Median (Q1, Q3)	43.3 (35.0, 54.3)	49.8 (38.3, 63.8)	42.8 (34.6, 53.5)	0.169
Follow-up time (months), Mean ± SD	54.6 ± 37.4	42.0 ± 35.6	58.8 ± 37.6	0.223
CIT (h), Median (Q1, Q3)	11.8 (8.0, 18.6)	11.0 (10.0, 12.5)	13.0 (8.0, 19.9)	0.521
Surgery method, n (%)				0.161
EBKT	33 (82)	10 (100)	23 (77)	
SDKT	7 (18)	0 (0)	7 (23)	
Induction, n (%)				1
ATG	32 (80)	8 (80)	24 (80)	
Basiliximab	8 (20)	2 (20)	6 (20)	
CNI, n (%)				1
FK506	40 (100)	10 (100)	30 (100)	
MPA, n (%)				0.189
Mycophenolate acid	15 (38)	6 (60)	9 (30)	
Mycophenolate mofetil	25 (62)	4 (40)	21 (70)	
Primary disease, n (%)				0.707
Glomerulonephritis	26 (65)	7 (70)	19 (63)	
IgA nephropathy	5 (12.5)	2 (20)	3 (10)	
Congenital or hereditary diseases	6 (15)	1 (10)	5 (17)	
Other	3 (7.5)	0 (0)	3 (10)	

BWHM, body weight high mismatch group; BWLM, body weight low mismatch group; EGFR, Estimated glomerular filtration rate; BWR, body weight ratio (donor/recipient); SD, standard deviation; CIT, cold ischemia time; EBKT, en-bloc kidney transplantation; SDKT, split dual kidney transplantation; ATG: antithymocyte globulin; CNI: calcineurin inhibitor; MPA: mycophenolic acid.

*According to the donor type standards published by Chinese scholars, the DCD referred to in this study includes Donation after Brain Death followed by Circulatory Death (DBCD), which differs from the broader definition of DCD used internationally [21].

### Patient and graft survival

**Figure 1.**
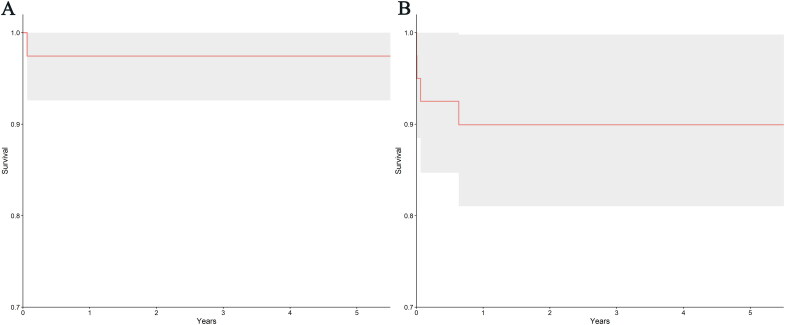
KM curves of patients and graft survival rates of 40 DKT recipients. (A) KM curves of patient survival rates of 40 DKT recipients. (B) KM curves of graft survival rates of 40 DKT recipients. DKT, dual kidney transplantation; KM, Kaplan-Meier.

The 1, 3, and 5-year patient survival rates for the 40 DKT recipients were 97.4% (83.2% to 99.6%). The 1, 3, and 5-year graft survival rates were 89.9% (ranging from 75.4% to 96.1%) (see Figure 1A and B). Among the recipients, one died of acute inferior myocardial infarction (1/40, 2.5%), while five patients experienced graft loss (5/40, 12.5%). This included two cases of renal vascular thrombosis, one case of ureteral stricture with urinary leakage, one case of mixed rejection, and one case of primary non-function (PNF) of the transplanted kidney. Additionally, one pediatric recipient suffered from primary non-function (PNF) after transplantation (1/40, 2.5%), possibly due to postoperative heart failure and unstable blood volume. However, this recipient underwent dual kidney removal and subsequent retransplantation, and currently, the graft function is good.

**Figure 2.**
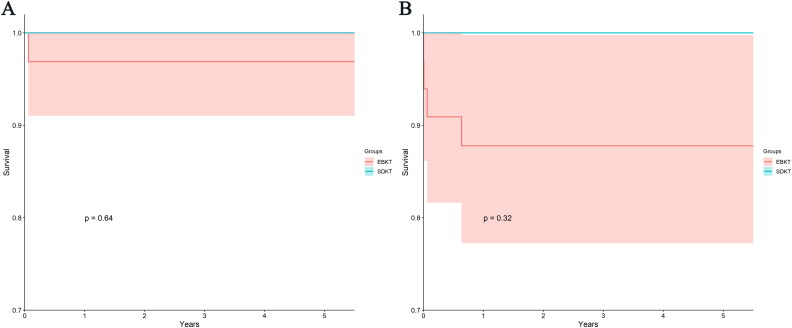
KM curves of patients and graft survival rates of EBKT group and SDKT group. (A) KM curves of patient survival rates of EBKT group and SDKT group. There was no difference between EBKT group and SDKT group in patient survival rates (*p* = 0.64). (B) KM curves of graft survival rates of EBKT group and SDKT group. The graft survival rates of EBKT group were similar to that of SDKT group (*p* = 0.32). KM, Kaplan-Meier; EBKT, en-bloc kidney transplantation; SDKT, split dual kidney transplantation.

**Figure 3.**
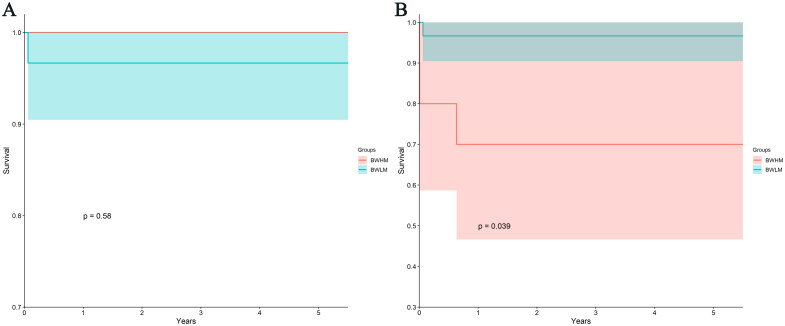
KM curves of patients and graft survival rates of BWHM group and BWLM group. (A) KM curves of patient survival rates of BWHM group and BWLM group. There was no difference between BWHM group and BWLM group in patient survival rates (*p* = 0.58). (B) KM curves of graft survival rates of BWHM group and BWLM group. The graft survival rates of BWLM group were significantly higher than that of BWHM group (*p* = 0.039). KM, Kaplan-Meier; BWHM, body weight high mismatch; BWLM, body weight low mismatch.

There was no statistically significant difference in patient survival between the EBKT and SDKT groups (*p* = 0.640) (see Figure 2A and B). The EBKT group had patient survival rates of 96.9% (79.8%, 99.6%) at 1, 2, 3, 4, and 5 years, while the SDKT group’s patient survival rates were 100% for the same time period. Furthermore, the EBKT group exhibited graft survival rates of 87.8% (70.6%, 95.2%) at 1, 2, 3, 4, and 5 years, while the SDKT group’s graft survival rates were 100% for the corresponding time intervals. Notably, there was no statistically significant contrast observed between the groups (*p* = 0.318). When stratifying the recipients into two groups based on the first quartile of the body weight ratio between donors and recipients, it was found that the BWHM group had patient survival rates of 100% at 1, 2, 3, 4, and 5 years, while the BWLM group had patient survival rates of 96.7% (78.6%, 99.5%) at the same time intervals. No statistically significant difference was observed between these two groups (*p* = 0.584, see Figure 3A). In terms of graft survival, the BWHM group exhibited graft survival rates of 70% (32.9%, 89.2%) at 1, 2, 3, 4, and 5 years, which were significantly lower compared to the rates observed in the BWLM group at 96.7% (78.6%, 99.5%) (*p* = 0.039, see Figure 3B).

**Figure 4.**
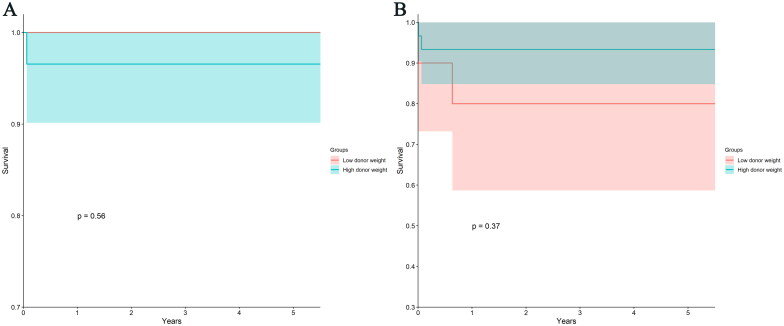
KM curves of patients and graft survival rates of low donor weight group and high donor weight group. (A) KM curves of patient survival rates of low donor weight group and high donor weight group. There was no difference between low donor weight group and high donor weight group in patient survival rates (*p* = 0.56). (B) KM curves of graft survival rates of low donor weight group and high donor weight group. The graft survival rates of low donor weight group were similar to that of high donor weight group (*p* = 0.37). KM, Kaplan-Meier.

To mitigate potential confounding factors arising from small donor body weights, we conducted a stratified analysis based on donor weight. We employed the same analytical strategy used for the donor-recipient body weight ratio, selecting the first quartile of overall donor weight (3.9 kg) as a threshold to categorize patients into a low donor weight group (*n* = 10) and a high donor weight group (*n* = 30). The patient survival rates at 1, 2, 3, 4, and 5 years were 96.7% (77.9%, 99.5%) in the high donor body weight group and 100% in the low donor body weight group. There was no statistically significant difference observed between the two groups (*p* = 0.557, see Figure 4A). Similarly, the graft survival rates at 1, 2, 3, 4, and 5 years were 93.3% (75.9%, 98.3%) in the high donor body weight group and 80.0% (40.9%, 94.6%) in the low donor body weight group. Once again, there was no statistically significant difference between the two groups (*p* = 0.365, see Figure 4B).

### Posttransplant complications

The postoperative complications of the 40 renal transplant recipients are detailed in Table 3. A total of nine recipients experienced delayed graft function (DGF) after DKT (9/40, 22.5%). This included five adult recipients (5/26, 19.2%) and four pediatric recipients (4/14, 28.6%). Among them, one adult recipient developed DGF and subsequently suffered bilateral vascular thrombosis, resulting in graft loss. This recipient underwent dual kidney removal but tragically succumbed to acute myocardial infarction. The other eight recipients who experienced DGF gradually regained normal graft function after undergoing temporary dialysis. Seventeen patients (17/40, 42.5%) experienced various types of infections. The most prevalent types were urinary tract infections (9/40, 22.5%) and pulmonary infections (8/40, 20%). All patients with infections recovered following targeted antimicrobial treatment. One adult recipient was a carrier of the hepatitis B virus pre-surgery and was subsequently diagnosed with liver cancer six years post-transplantation. This recipient underwent partial liver resection and exhibited excellent recovery with no abnormalities in kidney function.

**Table 3.** Complications grouped by surgical procedure.

Variables	Total (*n* = 40)	EBKT (*n* = 33)	SDKT (*n* = 7)	*P*
DGF, n (%)				0.645
Positive	9 (22)	7 (21)	2 (29)	
PNF, n (%)				1
Positive	1 (2)	1 (3)	0 (0)	
Pulmonary infections, n (%)				0.309
Positive	8 (20)	8 (24)	0 (0)	
Urinary infections, n (%)				1
Positive	9 (22)	8 (24)	1 (14)	
BKV infections, n (%)				1
Positive	1 (2)	1 (3)	0 (0)	
Other infections, n (%)				1
Positive	11 (28)	9 (27)	2 (29)	
Graft-related surgical complications, n (%)				0.309
Positive	8 (20)	8 (24)	0 (0)	
Urinary tract surgical complications, n (%)				1
Positive	4 (10)	4 (12)	0 (0)	
TCMR, n (%)				1
Positive	3 (8)	3 (9)	0 (0)	
ABMR, n (%)				1
Positive	0 (0)	0 (0)	0 (0)	
Mixed Rejection, n (%)				1
Positive	2 (5)	2 (6)	0 (0)	

EBKT, en-bloc kidney transplantation; SDKT, split dual kidney transplantation; DGF, delayed graft function; PNF, primary nonfunction; BKV, BK virus; TCMR, T cell-mediated rejection; ABMR, antibody mediated rejection.

A total of 8 DKT recipients (8/40, 20%) experienced graft-related surgical complications. Among them, 6 patients (6/40, 15%) encountered vascular thrombosis, with a median donor age of 3 months. Except for one donor aged 2 years, the remaining five donors had an average age of 2.1 months. Additionally, 2 recipients suffered from renal artery stenosis (2/40, 5%), and another recipient developed a hematoma in the transplanted kidney (1/40, 2.5%). Of the six recipients with vascular thrombosis, two experienced bilateral thrombosis, leading to graft loss. Besides the recipient who tragically succumbed to myocardial infarction due to bilateral vascular thrombosis, another recipient manifested oliguria post-transplantation. Surgical exploration revealed a purple appearance in both transplanted kidneys, with signs of rupture in the lower pole of the right kidney, indicating bilateral vascular thrombosis. Consequently, this recipient underwent bilateral graft removal. Subsequently, secondary kidney transplantation was performed, yielding stable functioning of the newly transplanted kidney.

Four recipients experienced unilateral vascular thrombosis, resulting in graft atrophy on the affected side for three of them. However, the contralateral side of the transplanted kidney remained unaffected, and the serum creatinine levels of these recipients are currently stable. Another recipient developed unilateral vascular thrombosis during the recovery period, accompanied by a ureteral fistula. Nonetheless, the serum creatinine level of this recipient is currently within the normal range.

Two recipients developed renal artery stenosis, with one case resulting in primary non-function (PNF), which required the removal of both transplanted kidneys and subsequent retransplantation. The other recipient presented with renal artery stenosis along with thrombosis in the transplanted kidney, resulting in atrophy on one side of the transplanted kidney, while the function of the contralateral transplanted kidney remained intact.

Lastly, one patient experienced a hematoma in the transplanted kidney, which was subsequently surgically removed. Unfortunately, this patient later developed a urinary fistula and obstruction in the urinary tract, necessitating the removal of both transplanted kidneys and a second transplant.

The postoperative outcomes of the EBKT and SDKT groups are presented in Table 3. With respect to postoperative complications, no substantial disparities were observed between the two groups. It is noteworthy that all 8 instances of graft-related surgical complications occurred within the EBKT cohort, although the difference between the two groups was not statistically significant (*p* = 0.880).

The postoperative complications of the BWLM group and BWHM group are outlined in Table 4. The incidence of graft-related surgical complications was significantly higher in the BWHM group compared to the BWLM group (50.0% vs. 6.7%, *p* = 0.022). Similarly, the occurrence of urinary tract surgical complications was significantly higher in the BWHM group compared to the BWLM group (30.0% vs. 10.0%, *p* = 0.042). There were no significant differences between the two groups in terms of other postoperative complications.

**Table 4.** Complications grouped by donor-recipient body weight ratio.

Variables	Total (*n* = 40)	BWHM (*n* = 10)	BWLM (*n* = 30)	*P*
DGF, n (%)				0.19
Positive	9 (22)	4 (40)	5 (17)	
PNF, n (%)				0.25
Positive	1 (2)	1 (10)	0 (0)	
Pulmonary infections, n (%)				1
Positive	8 (20)	2 (20)	6 (20)	
Urinary infections, n (%)				0.665
Positive	9 (22)	3 (30)	6 (20)	
BKV infections, n (%)				1
Positive	1 (2)	0 (0)	1 (3)	
Other infections, n (%)				0.418
Positive	11 (28)	4 (40)	7 (23)	
Graft-related surgical complications, n (%)				**0.022**
Positive	8 (20)	5 (50)	3 (10)	
Urinary tract surgical complications, n (%)				**0.042**
Positive	4 (10)	3 (30)	1 (3)	
TCMR, n (%)				1
Positive	3 (8)	1 (10)	2 (7)	
ABMR, n (%)				1
Positive	0 (0)	0 (0)	0 (0)	
Mixed Rejection, n (%)				1
Positive	2 (5)	0 (0)	2 (7)	

BWHM, body weight high mismatch group; BWLM, body weight low mismatch group; DGF, delayed graft function; PNF, primary nonfunction; BKV, BK virus; TCMR, T cell-mediated rejection; ABMR, antibody mediated rejection.

Table 5 illustrates the postoperative complications of the two cohorts stratified by donor body weight alone. The prevalence of urinary tract surgical complications in the transplanted kidneys was significantly higher in the low donor body weight cohort compared to the high donor body weight cohort (30.0% vs. 10.0%, *p* = 0.042). Nonetheless, no significant differences were observed in other facets of postoperative complications between the two cohorts.

**Table 5.** Complications grouped by donor body weight.

Variables	Total (*n* = 40)	Low donor weight (*n* = 10)	High donor weight (*n* = 30)	P
DGF, n (%)				0.19
Positive	9 (22)	4 (40)	5 (17)	
PNF, n (%)				0.25
Positive	1 (2)	1 (10)	0 (0)	
Pulmonary infections, n (%)				0.388
Positive	8 (20)	3 (30)	5 (17)	
Urinary infections, n (%)				0.19
Positive	9 (22)	4 (40)	5 (17)	
BKV infections, n (%)				1
Positive	1 (2)	0 (0)	1 (3)	
Other infections, n (%)				0.103
Positive	11 (28)	5 (50)	6 (20)	
Graft-related surgical complications, n (%)				0.089
Positive	8 (20)	4 (40)	4 (13)	
Urinary tract surgical complications, n (%)				**0.042**
Positive	4 (10)	3 (30)	1 (3)	
TCMR, n (%)				0.149
Positive	3 (8)	2 (20)	1 (3)	
ABMR, n (%)				1
Positive	0 (0)	0 (0)	0 (0)	
Mixed Rejection, n (%)	38 (95)	10 (100)	28 (93)	1
Positive	2 (5)	0 (0)	2 (7)	

DGF, delayed graft function; PNF, primary nonfunction; BKV, BK virus; TCMR, T cell-mediated rejection; ABMR, antibody mediated rejection.

### Renal function

The estimated glomerular filtration rate (eGFR) and levels of urinary protein between the EBKT and SDKT groups are presented in Table 6. The eGFR at postoperative years 1, 3, and 5 after DKT was (78.93 ± 25.23), (83.82 ± 32.4), and (85.92 ± 37.08) mL/min/1.73 m^2^, respectively. There were no statistically significant discrepancies observed in eGFR and proteinuria one year after the surgical procedures in the aforementioned groups. As evident from Table 6, a total of 18 recipients (18/37, 48.6%) demonstrated proteinuria, which either resolved or persisted as weak positivity following restoration of renal function or modification of antihypertensive regimen (angiotensin-converting enzyme inhibitors for blood pressure management) until the follow-up assessment. At the one-month postoperative evaluation, two patients experienced graft failure, and one recipient succumbed, thus necessitating their exclusion from the analysis.

**Table 6.** The serum creatinine and proteinuria status of the EBKT and SDKT groups.

Variables	Total (*n* = 40)	EBKT (*n* = 33)	SDKT (*n* = 7)	*P*
EGFR at 1-month (ml/min ·1.73 m^2^), Mean ± SD	46.3 ± 23.3	48.1 ± 23.6	39.1 ± 22.2	0.369
EGFR at 3-month (ml/min ·1.73 m^2^), Mean ± SD	58.1 ± 21.8	58.2 ± 20.0	57.7 ± 30.0	0.97
EGFR at 6-month (ml/min ·1.73 m^2^), Mean ± SD	69.1 ± 24.6	68.1 ± 21.1	73.3 ± 37.2	0.732
EGFR at 1-year (ml/min ·1.73 m^2^), Mean ± SD	78.9 ± 25.2	76.9 ± 22.6	87.0 ± 35.0	0.49
EGFR at 3-year (ml/min ·1.73 m^2^), Mean ± SD	83.8 ± 32.4	81.3 ± 30.7	105.1 ± 45.9	0.466
EGFR at 5-year (ml/min ·1.73 m^2^), Mean ± SD	85.9 ± 37.1	83.1 ± 36.0	100.0 ± 47.6	0.609
Urinary protein at 1-month, n (%)*				0.232
Positive	18 (49)	13 (43)	5 (71)	
Urinary protein at 3-month, n (%)*				1
Positive	8 (23)	7 (25)	1 (14)	
Urinary protein at 6-month, n (%)*				1
Positive	5 (15)	4 (15)	1 (14)	
Urinary protein at 1-year, n (%)*				0.584
Positive	6 (18)	4 (15)	2 (29)	

EBKT, en-bloc kidney transplantation; SDKT, split dual kidney transplantation; EGFR, Estimated glomerular filtration rate.

*Patients with positive urinary protein tests at postoperative follow-up visits at 1 month, 3 months, 6 months, and 1 year. Proteinuria was defined as urine protein levels ranging from ± to +++ as measured by our hospital’s laboratory.

The eGFR and urinary protein levels of BWHM and BWLM groups are illustrated in Table 7. Regarding postoperative eGFR and proteinuria, it was discerned that the eGFR at the six-month mark was notably lower in the BWHM group compared to the BWLM group (51.03 ± 23.07 vs. 74.71 ± 22.68, *p* = 0.026). Additionally, the incidence of proteinuria at three months was significantly higher in the BWHM group compared to the BWLM group (75.0% vs. 7.4%, *p* < 0.001). Nonetheless, comparable outcomes were observed between the two groups at other time intervals.

**Table 7.** The serum creatinine and proteinuria status of the BWHM and BWLM groups.

Variables	Total (*n* = 40)	BWHM (*n* = 10)	BWLM (*n* = 30)	*P*
EGFR at 1-month (ml/min ·1.73 m^2^), Mean ± SD	46.3 ± 23.3	30.3 ± 31.1	51.0 ± 18.7	0.108
EGFR at 3-month (ml/min ·1.73 m^2^), Mean ± SD	58.1 ± 21.8	43.9 ± 25.0	62.3 ± 19.4	0.086
EGFR at 6-month (ml/min ·1.73 m^2^), Mean ± SD	69.1 ± 24.6	51.0 ± 23.1	74.7 ± 22.7	**0.026**
EGFR at 1-year (ml/min ·1.73 m^2^), Mean ± SD	78.9 ± 25.2	61.0 ± 29.0	83.4 ± 22.6	0.093
EGFR at 3-year (ml/min ·1.73 m^2^), Mean ± SD	83.8 ± 32.4	84.9 ± 43.3	83.5 ± 30.0	0.946
EGFR at 5-year (ml/min ·1.73 m^2^), Mean ± SD	85.9 ± 37.1	108.4 ± 35.4	81.4 ± 36.9	0.319
Urinary protein at 1-month, n (%)*				0.447
Positive	18 (49)	5 (62)	13 (45)	
Urinary protein at 3-month, n (%)*				**< 0.001**
Positive	8 (23)	6 (75)	2 (7)	
Urinary protein at 6-month, n (%)*				0.072
Positive	5 (15)	3 (38)	2 (8)	
Urinary protein at 1-year, n (%)*				1
Positive	6 (18)	1 (17)	5 (19)	

BWHM, body weight high mismatch group; BWLM, body weight low mismatch group; EGFR, Estimated glomerular filtration rate.

*Patients with positive urinary protein tests at postoperative follow-up visits at 1 month, 3 months, 6 months, and 1 year. Proteinuria was defined as urine protein levels ranging from ± to +++ as measured by our hospital’s laboratory.

When classified based on the weight of the donor, Table 8 illustrates the postoperative eGFR and proteinuria status of the two cohorts. Concerning the eGFR of the transplanted kidney, the eGFR at the first month and the third month was substantially lower in the group with donors of lower body weight compared to the group with donors of higher body weight (28.41 ± 20.58 vs. 52.47 ± 21.16, *p* = 0.009; 43.6 ± 18.5 vs. 63.1 ± 20.9, *p* = 0.019). However, there were no statistically significant disparities in eGFR at subsequent time intervals. With respect to proteinuria, the prevalence of proteinuria at three months was considerably higher in the group with donors of lower body weight compared to the group with donors of higher body weight (67.8% vs. 7.7%, *p* = 0.01), yet no significant variations in proteinuria incidence were observed at other time points.

**Table 8.** The serum creatinine and proteinuria status grouped by weight.

Variables	Total (*n* = 40)	Low donor weight (*n* = 10)	High donor weight (*n* = 30)	*P*
EGFR at 1-month (ml/min 1.73 m^2^), Mean ± SD	46.3 ± 23.3	28.4 ± 20.6	52.5 ± 21.2	**0.009**
EGFR at 3-month (ml/min 1.73 m^2^), Mean ± SD	58.1 ± 21.8	43.6 ± 18.5	63.1 ± 20.9	**0.019**
EGFR at 6-month (ml/min 1.73 m^2^), Mean ± SD	69.1 ± 24.6	55.7 ± 25.7	74.0 ± 22.8	0.083
EGFR at 1-year (ml/min 1.73 m^2^), Mean ± SD	78.9 ± 25.2	62.1 ± 25.1	83.9 ± 23.5	0.051
EGFR at 3-year (ml/min 1.73 m^2^), Mean ± SD	83.8 ± 32.4	74.0 ± 26.1	87.1 ± 34.2	0.309
EGFR at 5-year (ml/min 1.73 m^2^), Mean ± SD	85.9 ± 37.1	79.0 ± 32.5	87.3 ± 38.8	0.721
Urinary protein at 1-month, n (%)*				0.269
Positive	18 (49)	6 (67)	12 (43)	
Urinary protein at 3-month, n (%)*				**0.001**
Positive	8 (23)	6 (67)	2 (8)	
Urinary protein at 6-month, n (%)*				0.102
Positive	5 (15)	3 (33)	2 (8)	
Urinary protein at 1-year, n (%)*				1
Positive	6 (18)	1 (14)	5 (19)	

EGFR, Estimated glomerular filtration rate.

*Patients with positive urinary protein tests at postoperative follow-up visits at 1 month, 3 months, 6 months, and 1 year. Proteinuria was defined as urine protein levels ranging from ± to +++ as measured by our hospital’s laboratory.

## Discussion

Performing kidney transplantation from pediatric donors presents a notably more challenging surgical procedure, with an increased risk of hyperfiltration injury to the graft and a higher incidence of surgical complications, including vascular and urological issues. As a result, pediatric donors are frequently categorized as "marginal donors" in comparison to adult donors. Given the challenges and complications associated with kidney transplantation from pediatric donors, there has been substantial interest in investigating alternative techniques, such as DKT. DKT is regarded as a "supplementary option" to the standard SKT procedure, thereby aiding in the expansion of the donor pool [22–24]. Consequently, transplant centers worldwide have increasingly adopted DKT, with many studies reporting favorable long-term outcomes [12–15,25–30]. In our study, the donors’ body weights ranged from 2 kg to 15 kg. The overall graft survival rates at 1, 3, and 5 years post-transplantation for recipients who underwent DKT from pediatric deceased donors were 89.9%, aligning with findings from prior research. Our data indicate relatively satisfactory long-term graft survival in DKT, advocating for the expansion of the donor pool through DKT utilization. Further investigation revealed no significant overall difference between the two types of dual kidney transplantation procedures. Additionally, we identified a critical determinant impacting transplant outcomes: the donor-recipient body weight ratio (BWR). Complications, affecting renal function and graft survival, may arise in pairs with a large body weight difference (BWR > 1:10), contrasting with pairs with BWR < 1:10. Notably, this study is the first to propose donor-to-recipient weight ratios for pediatric donor kidney transplantation (DKT) within a cohort that includes both adult and pediatric recipients.

Several transplantation centers have reported noteworthy 2-year patient and graft survival rates following DKT from pediatric donors, reaching up to 97% and 94%, respectively. Furthermore, these transplanted kidneys have demonstrated enduring renal function, maintaining an estimated glomerular filtration rate (eGFR) above 60 mL/min/1.73 m^2^ even at 10 and 20 years post-transplantation [30,31]. Another investigation delineated that for pediatric recipients receiving kidneys from donors weighing between 5 to 10 kg, the incidence of graft loss within the first post-transplantation year resembles that of single kidney transplantation (SKT) from expanded criteria donors. Nonetheless, the long-term prognosis appears more favorable for DKT within this specific cohort [5]. These meticulous inquiries provide robust evidence advocating for the utilization of kidneys procured from pediatric donors, thereby bolstering the argument for their continued inclusion in organ transplantation endeavors and corroborating the conclusions drawn from our own investigative efforts.

Currently, there is no consensus regarding the utilization of kidneys from pediatric donors for DKT and the improvement of prognosis. In 2023, guidelines from Australia and New Zealand suggested that pediatric donors aged above 5 years or weighing above 20 kg could be considered equivalent to adult donors in allocation. However, for donors weighing between 10 kg and 20 kg, or aged between 1 and 5 years, priority should be given to performing EBKT. In cases where the donor weight is less than 10 kg, it is recommended to utilize EBKT, with a strict requirement for the surgical team to possess specialized surgical expertise and extensive experience [32]. The experience of utilizing donor kidneys weighing less than 5 kg is extremely rare, and at times even controversial. Indeed, the utilization rate of donor kidneys often decreases with decreasing donor age in transplant centers worldwide [22,28,33]. One factor contributing to the increasingly severe challenges is the growing complexity of donor-recipient matching. Other transplantation centers have placed increasing emphasis on donor-recipient matching, and some studies suggest a correlation between a low donor body weight to recipient body weight (DBW/RBW) ratio and reduced eGFR [17]. A study on a cohort of 20 adult dual-kidney recipients found that a donor-to-recipient weight ratio above 7.5 had short-term impacts on kidney function [16]. Analysis of SRTR data indicates that when the recipient exceeds the donor by more than 30 kg, the greatest relative hazards for graft failure were evident, particularly among female recipients of male donor kidneys and male recipients of female donor kidneys [19]. Additional studies have also demonstrated that a size mismatch between the donor and recipient could result in artery stenosis, and the use of adequate Carrel patches might mitigate this occurrence [18]. Similarly, at our center, a smaller disparity in donor body weight correlates with a significantly higher graft survival among recipients. Moreover, there is a notable decrease in postoperative vascular complications, providing additional support for the validity of this theory. The adverse effects of donor-recipient body weight mismatches on graft survival can generally be attributed to several plausible mechanisms. Based on our study findings and clinical experience, we hypothesize that potential mechanisms may include mismatches in the diameters of donor and recipient renal arteries and veins, as well as increased effective circulating blood volume in recipients with higher body weight. Hemodynamic changes associated with transitioning from small kidneys into large recipients may result in proteinuria and hypertension, leading to damage to the transplanted kidney. Furthermore, these compensatory changes may act as pro-inflammatory triggers, contributing to alloantigen-dependent renal injury. The use of calcineurin inhibitors in these patients, coupled with their potential nephrotoxicity and high-filtering hemodynamics (hyperfiltration syndrome), may adversely affect graft functions, future studies are needed to validate our hypotheses [16,34–36].

DKT from pediatric deceased donors presents significant challenges for transplant teams, and the overall efficacy may vary across different transplant centers. Even experienced transplant centers may experience postoperative complications and graft loss in DKT cases. Vascular thrombosis has been identified as a primary cause of graft loss after DKT, with studies indicating graft loss rates of 10%-25% among recipients of kidneys from donors aged 2 years or younger. Recipients of kidneys from donors aged less than 12 months are at increased risk of graft loss, with vascular thrombosis playing a significant contributory role [37,38]. Data from our center reveals an overall incidence of vascular thrombosis of 15.0% (6/40) in DKT recipients, with 4 cases involving thrombosis in a single kidney that did not result in complete loss of both grafts. Furthermore, among the donors corresponding to recipients who experienced thrombosis, 5 cases involved donors aged less than 5 months, with an incidence rate of 23.8% (5/21), while only 1 case of thrombosis occurred among recipients corresponding to donors aged over 5 months, with an incidence rate of 5.26% (1/19). This suggests that special attention should be directed toward the occurrence of thrombotic complications postoperatively when utilizing kidneys from donors aged less than 5 months. However, the overall incidence of thrombosis in our cohort is relatively low.

When it comes to preventing and managing surgical complications, it is crucial to recognize the unique characteristics of each surgical procedure. The EBKT procedure, characterized by its distinctive "common channel" design, is associated with complications such as vascular stenosis and anastomotic-related issues. These complications often result from insufficient protection of the donor’s arteries and veins during procurement and trimming, as well as imprecise manipulation of the anastomotic sites during transplantation. In our center, the overall incidence of vascular anastomotic complications after DKT is 5% (2/40), a rate considered acceptable compared to that of other centers [13,30,31]. To mitigate such complications, our center typically utilizes the donor’s aorta and inferior vena cava as the "common channel" in the EBKT procedure. The main vascular trunk of the "common channel" is more appropriately sized to match the recipient’s vessels, and the larger anastomotic area significantly reduces the risk of postoperative stenosis or occlusion. When performing the SDKT procedure, a combination of continuous and interrupted suturing techniques may be employed for the arterial anastomosis of the dual kidneys to minimize the risk of anastomotic stenosis. During the trimming process of the dual kidney in our center, we strive to avoid re-perfusion or detect arterial and venous leakage, aiming to prevent vascular intimal injury that may lead to stenosis. Regarding proteinuria, multiple studies have reported the risk of insufficient effective nephron units in kidneys from pediatric donors, leading to long-term glomerular hyperfiltration and early-onset proteinuria. In our cohort, recipients experienced a gradual reduction in proteinuria within the first year post-transplant, with no statistically significant difference observed between surgical groups. This indirectly supports the assertion that the number of effective nephron units in full-term neonatal kidneys is equivalent to that of adults, as well as the concept of the high adaptability of pediatric kidneys. In clinical practice, it is essential to emphasize the importance of blood pressure control in these recipients during the early post-transplant period.

However, this article has several limitations, including a small sample size, a single-center design, a retrospective methodology, and the absence of long-term follow-up data. Despite collecting a broad range of donor and recipient indicators, we cannot rule out unconsidered confounding factors. The small cohort may limit the detection of significant confounders and affect the reliability and generalizability of the findings. Thus, these results should be interpreted cautiously, and further research is needed to confirm them.

## Conclusions

In this study, we investigated the prognosis and safety of DKT in pediatric donors. The prospect for the DKT procedure, including both EBKT and SDKT, is highly promising, making it a reliable technique for expanding the pool of potential donors. When performing donor-recipient matching, avoiding pairings with significant differences in body weight may enhance early postoperative renal function recovery and long-term kidney viability in recipients.

## Supplementary Material

Supplementary_3nd revision.docx
