# Supplementary material for: The effect of low donor-to-recipient body weight ratio on graft survival after dual kidney transplantation from pediatric deceased donors
Source: Ren Fail. 2025 Jan 22;47(1):2454968. doi: 10.1080/0886022X.2025.2454968 (PMC11755734; doi:10.1080/0886022X.2025.2454968)
Supplement: Supplementary_3nd revision.docx [file IRNF_A_2454968_SM4201.docx]

**Supplementary**

1. **If recipients were grouped by the median of donor-recipient body weight ratio (0.14).**


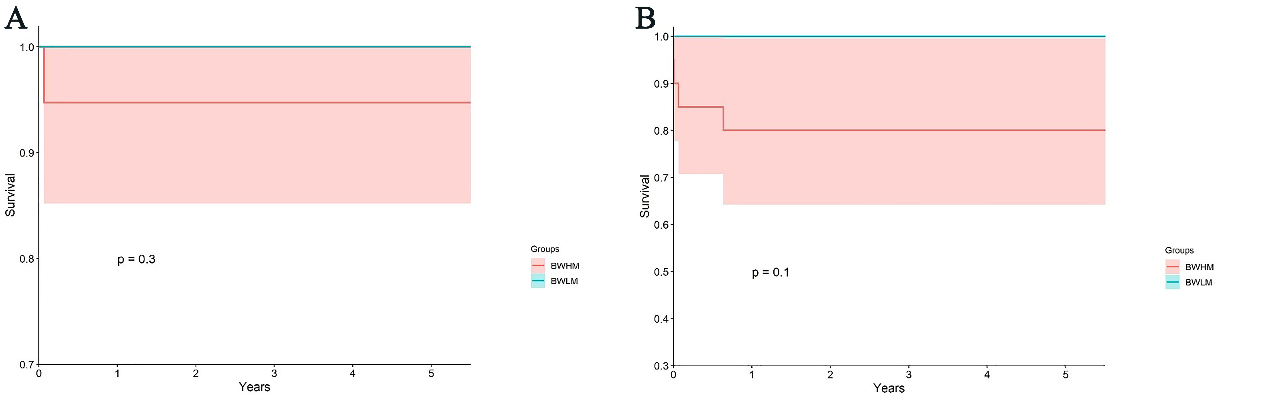


**Figure S1|** KM curves of patients and graft survival rates of BWHM group (n = 20) and BWLM group (n = 20). (If recipients were grouped by the median of donor-recipient body weight ratio). KM, Kaplan-Meier; BWHM, body weight high mismatch; BWLM, body weight low mismatch.

**TABLE S1|** Complications grouped by donor-recipient body weight ratio. (If recipients were grouped by the median of donor-recipient body weight ratio).

| **Variables** | **Total**  **(n = 40)** | **BWHM**  **(n = 20)** | **BWLM**  **(n = 20)** | ***P*** |
| --- | --- | --- | --- | --- |
| DGF, n (%) |  |  |  | 0.127 |
| Positive | 9 (22) | 7 (35) | 2 (10) |  |
| PNF, n (%) |  |  |  | 1 |
| Positive | 1 (2) | 1 (5) | 0 (0) |  |
| Pulmonary infections, n (%) |  |  |  | 0.695 |
| Positive | 8 (20) | 3 (15) | 5 (25) |  |
| Urinary infections, n (%) |  |  |  | 1 |
| Positive | 9 (22) | 5 (25) | 4 (20) |  |
| BKV infections, n (%) |  |  |  | 1 |
| Positive | 1 (2) | 0 (0) | 1 (5) |  |
| Other infections, n (%) |  |  |  | 0.479 |
| Positive | 11 (28) | 7 (35) | 4 (20) |  |
| Graft-related surgical complications, n (%) |  |  |  | 0.235 |
| Positive | 8 (20) | 6 (30) | 2 (10) |  |
| Urinary tract surgical complications, n (%) |  |  |  | 0.605 |
| Positive | 4 (10) | 3 (15) | 1 (5) |  |
| TCMR, n (%) |  |  |  | 1 |
| Positive | 3 (8) | 2 (10) | 1 (5) |  |
| ABMR, n (%) |  |  |  | 1 |
| Positive | 0 (0) | 0 (0) | 0 (0) |  |
| Mixed Rejection, n (%) |  |  |  | 0.487 |
| Positive | 2 (5) | 0 (0) | 2 (10) |  |

*BWHM, body weight high mismatch group; BWLM, body weight low mismatch group; DGF, delayed graft function; PNF, primary nonfunction; BKV, BK virus; TCMR, T cell-mediated rejection; ABMR, antibody mediated rejection.*

**TABLE S2|** The serum creatinine and proteinuria status of the BWHM and BWLM groups. (If recipients were grouped by the median of donor-recipient body weight ratio).

| **Variables** | **Total**  **(n = 40)** | **BWHM**  **(n = 10)** | **BWLM**  **(n = 30)** | ***P*** |
| --- | --- | --- | --- | --- |
| EGFR at 1-month (ml/min ·1.73 m^2^), Mean ± SD | 46.3 ± 23.3 | 40.4 ± 26.4 | 51.9 ± 19.1 | 0.151 |
| EGFR at 3-month (ml/min ·1.73 m^2^), Mean ± SD | 58.1 ± 21.8 | 55.1 ± 25.2 | 60.8 ± 18.4 | 0.451 |
| EGFR at 6-month (ml/min ·1.73 m^2^), Mean ± SD | 69.1 ± 24.6 | 66 ± 29.1 | 72.3 ± 19.5 | 0.464 |
| EGFR at 1-year (ml/min ·1.73 m^2^), Mean ± SD | 78.9 ± 25.2 | 76.5 ± 27.5 | 80.9 ± 23.8 | 0.621 |
| EGFR at 3-year (ml/min ·1.73 m^2^), Mean ± SD | 83.8 ± 32.4 | 93.4 ± 33.9 | 74.2 ± 28.8 | 0.12 |
| EGFR at 5-year (ml/min ·1.73 m^2^), Mean ± SD | 85.9 ± 37.1 | 96.7 ± 39.8 | 79.1 ± 35.4 | 0.361 |
| Urinary protein at 1-month, n (%)^*^ |  |  |  | 0.141 |
| Positive | 18 (49) | 11 (65) | 7 (35) |  |
| Urinary protein at 3-month, n (%)^*^ |  |  |  | **0.018** |
| Positive | 8 (23) | 7 (41) | 1 (6) |  |
| Urinary protein at 6-month, n (%)^*^ |  |  |  | 1 |
| Positive | 5 (15) | 3 (18) | 2 (12) |  |
| Urinary protein at 1-year, n (%)^*^ |  |  |  | 0.665 |
| Positive | 6 (18) | 2 (13) | 4 (22) |  |

*BWHM, body weight high mismatch group; BWLM, body weight low mismatch group; EGFR, Estimated glomerular filtration rate.*

^*^: Patients with positive urinary protein tests at postoperative follow-up visits at 1 month, 3 months, 6 months, and 1 year. Proteinuria was defined as urine protein levels ranging from +/- to +++ as measured by our hospital's laboratory.

1. **If recipients were grouped by the third quartile of donor-recipient body weight ratio (0.22).**


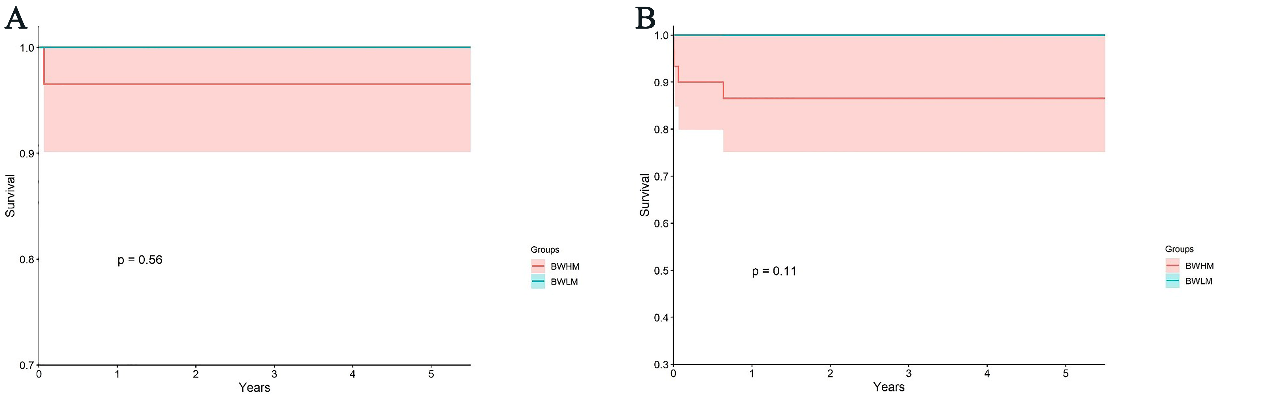


**Figure S2|** KM curves of patients and graft survival rates of BWHM group (n = 30) and BWLM group (n = 10). (If recipients were grouped by the third quartile of donor-recipient body weight ratio). KM, Kaplan-Meier; BWHM, body weight high mismatch; BWLM, body weight low mismatch.

**TABLE S3|** Complications grouped by donor-recipient body weight ratio. (If recipients were grouped by the third quartile of donor-recipient body weight ratio).

| **Variables** | **Total**  **(n = 40)** | **BWHM**  **(n = 30)** | **BWLM**  **(n = 10)** | ***P*** |
| --- | --- | --- | --- | --- |
| DGF, n (%) |  |  |  | 0.404 |
| Positive | 9 (22) | 8 (27) | 1 (10) |  |
| PNF, n (%) |  |  |  | 1 |
| Positive | 1 (2) | 1 (3) | 0 (0) |  |
| Pulmonary infections, n (%) |  |  |  | 0.388 |
| Positive | 8 (20) | 5 (17) | 3 (30) |  |
| Urinary infections, n (%) |  |  |  | 0.404 |
| Positive | 9 (22) | 8 (27) | 1 (10) |  |
| BKV infections, n (%) |  |  |  | 0.25 |
| Positive | 1 (2) | 0 (0) | 1 (10) |  |
| Other infections, n (%) |  |  |  | 0.696 |
| Positive | 11 (28) | 9 (30) | 2 (20) |  |
| Graft-related surgical complications, n (%) |  |  |  | 0.653 |
| Positive | 8 (20) | 7 (23) | 1 (10) |  |
| Urinary tract surgical complications, n (%) |  |  |  | 0.556 |
| Positive | 4 (10) | 4 (13) | 0 (0) |  |
| TCMR, n (%) |  |  |  | 1 |
| Positive | 3 (8) | 2 (7) | 1 (10) |  |
| ABMR, n (%) |  |  |  | 1 |
| Positive | 0 (0) | 0 (0) | 0 (0) |  |
| Mixed Rejection, n (%) |  |  |  | 1 |
| Positive | 2 (5) | 2 (7) | 0 (0) |  |

*BWHM, body weight high mismatch group; BWLM, body weight low mismatch group; DGF, delayed graft function; PNF, primary nonfunction; BKV, BK virus; TCMR, T cell-mediated rejection; ABMR, antibody mediated rejection.*

**TABLE S4|** The serum creatinine and proteinuria status of the BWHM and BWLM groups. (If recipients were grouped by the third quartile of donor-recipient body weight ratio).

| **Variables** | **Total**  **(n = 40)** | **BWHM**  **(n = 10)** | **BWLM**  **(n = 30)** | ***P*** |
| --- | --- | --- | --- | --- |
| EGFR at 1-month (ml/min ·1.73 m^2^), Mean ± SD | 46.3 ± 23.3 | 46 ± 24.7 | 47.2 ± 20 | 0.882 |
| EGFR at 3-month (ml/min ·1.73 m^2^), Mean ± SD | 58.1 ± 21.8 | 57.6 ± 22 | 59.5 ± 22.5 | 0.829 |
| EGFR at 6-month (ml/min ·1.73 m^2^), Mean ± SD | 69.1 ± 24.6 | 68.1 ± 25.3 | 72 ± 23.7 | 0.681 |
| EGFR at 1-year (ml/min ·1.73 m^2^), Mean ± SD | 78.9 ± 25.2 | 80.1 ± 24.4 | 75.9 ± 28.3 | 0.686 |
| EGFR at 3-year (ml/min ·1.73 m^2^), Mean ± SD | 83.8 ± 32.4 | 89.1 ± 34.9 | 70.6 ± 21.3 | 0.101 |
| EGFR at 5-year (ml/min ·1.73 m^2^), Mean ± SD | 85.9 ± 37.1 | 89.6 ± 38.5 | 78.6 ± 36.3 | 0.568 |
| Urinary protein at 1-month, n (%)^*^ |  |  |  | 0.714 |
| Positive | 18 (49) | 14 (52) | 4 (40) |  |
| Urinary protein at 3-month, n (%)^*^ |  |  |  | 0.648 |
| Positive | 8 (23) | 7 (27) | 1 (11) |  |
| Urinary protein at 6-month, n (%)^*^ |  |  |  | 1 |
| Positive | 5 (15) | 4 (16) | 1 (11) |  |
| Urinary protein at 1-year, n (%)^*^ |  |  |  | 0.336 |
| Positive | 6 (18) | 3 (13) | 3 (30) |  |

*BWHM, body weight high mismatch group; BWLM, body weight low mismatch group; EGFR, Estimated glomerular filtration rate.*

^*^: Patients with positive urinary protein tests at postoperative follow-up visits at 1 month, 3 months, 6 months, and 1 year. Proteinuria was defined as urine protein levels ranging from +/- to +++ as measured by our hospital's laboratory.
